# Recent Progress in the Diagnosis and Treatment of Melanoma and Other Skin Cancers

**DOI:** 10.3390/cancers15061824

**Published:** 2023-03-17

**Authors:** Laura Pawlik, Sarah Morgenroth, Reinhard Dummer

**Affiliations:** Dermatology Department, University Hospital Zurich and Medical Faculty, University of Zurich, 8091 Zurich, Switzerland

In this Special Issue, the reader will find nine papers regarding recent progress in diagnosis and treatment to optimize the clinical management of melanoma and non-melanoma skin cancer.

Early detection and resection of cutaneous melanoma are essential for a thorough prognosis [1]. A current topic regarding melanoma diagnosis is delayed diagnosis in the context of the COVID-19 lockdown. Kostner et al., 2022 report that in Switzerland, only older female patients showed a greater tumor thickness at first diagnosis during lockdown compared to pre- and post-lockdown periods [2]. The diagnostic delay in the subgroup of elderly females is attributed to gender-specific fearful and reserved attitudes during the COVID-19 pandemic. While skin self-exams remain the backbone of melanoma prevention, a short time interval dedicated to professional assessment is crucial to avoid upstaging. 

However, the visual differentiation of early melanoma from benign nevi remains difficult. Often, the patient and their families first recognize melanoma [3]. Therefore, smartphone applications based on artificial intelligence (AI) that support skin self-exams are becoming increasingly popular [4]. Jahn et al., 2022 investigated the diagnostic accuracy of the SkinVision^®^ application in classifying pigmented lesions as benign or possible melanoma [5]. The sensitivity and specificity in the differentiation of nevi and melanoma were low, with 41.3–83.3% and 60.0–82.9%, respectively. Therefore, applications such as these may exacerbate the current situation of over-diagnosis, in which a rising incidence of melanoma is not accompanied by rising mortality [6]. Laura K. Ferris reports: “*The absence of a coordinated strategy and guidelines results in largely patient-driven opportunistic screening with overrepresentation of those with a higher socioeconomic status. It is important to identify, educate, and, when appropriate, screen those most likely to die of melanoma and least likely to find their own melanomas at a treatable stage. In addition, efforts aimed at melanoma prevention are likely to be more effective and less costly at a population level than early detection through screening*.” [7].

Modern non-invasive diagnostic tools for skin cancer diagnosis also include optical coherence tomography (OCT) and reflectance confocal microscopy (RCM). Schuh et al., 2022 describe the differentiation of nevi and melanoma using line-field confocal optical coherence tomography (LC-OCT), a new technology with high penetration depths and high single-cell resolution similar to RCM [8]. By non-invasively differentiating nevi and melanoma using LC-OCT, the frequency of unnecessary surgical procedures could be reduced.

Another non-invasive strategy in melanoma staging and risk stratification is circulating tumor DNA (ctDNA). DNA is released during tissue remodeling and cell death; after entering the bloodstream, cell-free DNA is called ctDNA [9]. Established staging methods in melanoma to guide clinical risk stratification and decision-making are Breslow tumor thickness, sentinel lymph node biopsy (SLNB), ultrasound and PET-CT scans, and to a lesser extent, serum markers such as S-100. Boerlin et al., 2022 postulate that ctDNA could be used as a new tool for monitoring and predicting disease progression and therapeutic response. CtDNA, or “liquid biopsies”, could also aid in molecular analysis to detect tumor-specific genomic variants [10].

Progress has also been made in rare forms of melanoma. Argininosuccinate synthase 1 activity is a typical metabolic feature of uveal melanoma and the potential therapeutic target. Kraehenbuehl et al., 2022 investigated the effect of triple therapy, consisting of a combination of immunotherapy and pegylated arginine deiminase (ADI-PEG 20) [11]. Despite a solid tolerability and safety profile, no clinical benefit was demonstrated.

Immunotherapy with immune checkpoint inhibitors has revolutionized the treatment of melanoma patients [12]. However, as with any cancer treatment, immune checkpoint inhibitors are associated with a range of potential side effects, collectively referred to as immune-related adverse events (irAEs). While immunotherapy has significantly improved patient outcomes, irAEs remain a significant concern, with immune-related colitis being one of the most common reasons for therapy discontinuation. Kuo et al., 2022 note that gastrointestinal immune-related adverse events (GI irAEs) often appear after cutaneous irAEs [13]. They postulate that communication via the gut–skin axis could play a role in this relationship mechanism.

A common delayed adverse event is vitiligo-like depigmentation (VLD), observed after long-term treatment with immunotherapy. VLD is strongly associated with improved overall survival and progression-free survival, independent of other prognostic factors [14]. Hermann et al., 2022 observed that the upregulation of EDAR and downregulation of LAG3 was seen in responders amongst melanoma patients with VLD [15].

The advances in non-melanoma skin cancer treatment are also notable. A review of recent improvements in the diagnosis and management of high-risk SCC emphasizes the importance of classification in low and high-risk patients using specific criteria [16]. This classification supports therapy decision-making, as only a minority of SCC patients develop metastases and can profit from adjuvant systemic treatment.

In a patient cohort with locally advanced and metastatic basal cell carcinoma (BCC) requiring systemic treatment, a retrospective analysis of the efficacy and side effects of Smoothened inhibitors was performed by Grossmann et al., 2022 [17]. The patients in their study represented a more “real-life” population with extensive comorbidities that would typically prohibit inclusion in clinical trials. They found that smoothened inhibitors’ typical adverse event spectrum included decreased muscle spasms and alopecia, limiting treatment duration and long-term disease control. Intermittent dosing is a simple and valuable tool to postpone treatment discontinuation due to the discomfort of the medication. Overall, the study provides valuable information on using smoothened inhibitors in a patient population that may be underrepresented in clinical trials.

This collection of papers is an important contribution to our practical management of skin cancer patients in specialized units. 
